# Evaluating dissemination strategies to promote preteen HPV vaccination

**DOI:** 10.1186/1748-5908-10-S1-A5

**Published:** 2015-08-14

**Authors:** Joan R Cates, Sandra J Diehl, Jamie L Crandell, Tamera Coyne-Beasley

**Affiliations:** School of Journalism and Mass Communication, University of North Carolina at Chapel Hill, University of North Carolina, Chapel Hill, NC 27599-3365 USA; NC TraCS Institute, Community Academic Resources for Engaged Scholarship (CARES), Home of the UNC Clinical and Translational Science Awards (CTSA), University of North Carolina, Chapel Hill, NC 27599-7064 USA; School of Nursing and Department of Biostatistics, 2011 Carrington Hall, University of North Carolina at Chapel Hill, Chapel Hill, NC 27599-7460 USA; NC Child Health Research Network, Community Engagement NC TraCS Institute - Child Health Core Home of the UNC-CH Clinical and Translational Science Awards (CTSA); Division of General Pediatrics and Adolescent Medicine, University of North Carolina, Chapel Hill, NC 27599 USA

## Introduction

Adoption of vaccination against human papillomavirus (HPV) has been slow in the US. Routine HPV vaccination of 11-12 year old boys was recommended by CDC in late 2011, five years after the recommendation for routine vaccination was issued for girls. We developed and evaluated a social marketing intervention with parents and healthcare providers to stimulate adoption for preteen boys.

## Methods

Our intervention promoted HPV vaccine with both parents of preteen boys and health providers in 13 south central NC counties, July-September, 2012. The 'Protect Him' campaign included distribution of posters and brochures (English and Spanish) to county health departments (n = 13) plus 184 providers, two radio PSAs, a one hour online training for providers and a website. To assess changes in awareness, knowledge, attitudes, beliefs and vaccination actions, we evaluated the campaign using: 1) two independent, cross-sectional telephone surveys of parents with 9-13 year old boys (pre n = 516, post n = 455); 2) pre- and post-intervention surveys with enrolled providers; and 3) NC Immunization Registry (NCIR) data to compare vaccination rates in the 13 intervention vs. 14 control counties.

## Findings

Post intervention, providers reported increased likelihood of discussing (p=.03), recommending (p=.004) and vaccinating (P < .0001) 11-12 year old boys against HPV. Parents with campaign recall (62%; n = 284) were more likely to have heard of the HPV virus and vaccine (adjusted OR = 5.44 and 9.70 respectively, P < .0001). A Cox proportional hazards model for the NCIR data showed an intervention effect (HR = 1.34, p=.0024), as the probability of vaccination increased by 34% in the intervention counties relative to control counties during the three months of the intervention.

## Implications of research

This focused, time-limited, low intensity dissemination intervention boosted preteen HPV vaccination. Multiple evaluation strategies provide insight into intervention impact and potential explanatory mechanisms.

## Funding

This study was supported by grants from the National Institutes of Health

1R21A1095590-01A1 (Cates PI), and by the North Carolina Translational and Clinical Sciences Institute, through support from the National Center for Advancing Translational Sciences (NCATS), National Institutes of Health, Grant Award Number 1UL1TR001111. The content is solely the responsibility of the authors and does not necessarily represent the official views of the NIH.

